# Is susceptibility to severe COVID‐19 disease an inborn error of metabolism?

**DOI:** 10.1002/jimd.12280

**Published:** 2020-07-15

**Authors:** Peter T. Clayton

**Affiliations:** ^1^ Genetics and Genomic Medicine UCL Great Ormond Street Institute of Child Health London UK

At the time of writing, severe Covid‐19 disease has been responsible for 383 000 deaths worldwide (Johns Hopkins data). Most deaths occur in the <10% of infected individuals that develop respiratory failure.^1^ This is caused by bilateral interstitial pneumonia and acute respiratory distress syndrome and leads to dependence on mechanical ventilation.^2^ The pathogenesis of the pneumonia is poorly understood although the identification of a lymphocytic endotheliitis and widespread microvascular and macrovascular thromboembolic complications have led to the suggestion that the virus primarily injures the vascular endothelium.^3^, ^4^, ^5^ Patients with severe disease have high levels of proinflammatory cytokines.^6^, ^7^, ^8^ An important driver of cytokine production in viral infections as well as in autoimmune disorders is the unfolded protein response in the endoplasmic reticulum.^9^

Ellinghaus et al recently reported a genome‐wide association analysis comparing patients with respiratory failure in the SARS‐CoV‐2 pandemic in Italy and Spain to population derived controls.^10^ They detected cross‐replicating associations with rs11385942 at chromosome 3p21.31 and rs657152 at 9q34, which were genome‐wide significant (*P* < 5 × 10^−8^). Among the six genes at 3p21.31 was one, *SLC6A20*, which encodes the proline transporter SIT1 that is mutated in hyperglycinuria and iminoglycinuria.^11^ Ellinghaus et al suggested that the importance of SIT1 is because it interacts with angiotensin converting enzyme 2 (ACE), the SARS‐CoV‐2 cell receptor.^10^, ^12^, ^13^ However, it could be the protein's function in transporting proline that is important in severe Covid‐19 disease.

Proline is an important intracellular osmolyte with profound effects on protein folding.^14^, ^15^ Over‐expression of a cell surface proline transporter in *Escherichia coli* can prevent misfolding of a model protein and of constructs containing exon 1 of huntingtin with extended polyamine tracts. Other osmolytes have also been shown to ameliorate defective protein folding. For example, trimethylamine N‐oxide (TMAO) corrects assembly defects of branched chain α‐ketoacid decarboxylase in maple syrup urine disease,^16^ reverses defective trafficking of the ΔF508 cystic fibrosis transmembrane conductance regulator in cystic fibrosis,^17^ and enhances antigen presentation in antigen‐presenting cells.^18^

So, it is possible that low activity of SIT1 leads to a low proline concentration at sites of protein folding, leading to an exaggerated unfolded protein response in helper T‐lymphocytes and macrophages, thereby contributing to a pathogenic cytokine storm. Alternatively, higher than normal SIT1 activity could lead to unusually high‐proline concentrations impairing the immune response. Either mechanism could contribute to the increased morbidity and mortality. This hypothesis can be tested by measuring the proline content of peripheral blood mononuclear cells in individuals with good and poor outcomes of Covid‐19 infection. Plasma and urine concentrations of proline may also be different in the two groups. Genome sequencing projects will be able to confirm that there are specific variants in *SLC6A20* associated with poor outcome; it will be important to use expression studies to determine whether they are gain‐of‐function or loss‐of‐function variants. There may be a therapeutic role for dietary proline supplementation or restriction.

This article does not contain any studies with human or animal subjects performed by the author.
